# Acephalous Fœtus, Living Eight Hours

**Published:** 1833

**Authors:** 


### V. Acephalous Fœtus, living eight hours.

Dr. Jonathan Leatherman, of Cannonsburg, Pennsylvania, has communicated to us the following account of a *lusus naturæ*:

"A most singular exhibition of the aberrations of nature, occurred a few days since in the course of my obstetrical practice. Mrs. C., a woman of a healthy constitution, free from predisposition to any disease, was taken with labour pains, having, as she thought, exceeded her time *two months*, and was delivered of a female child, which presented the following peculiarities: That portion of the cranium which contains the cerebrum, was entirely wanting; and that which encloses the cerebellum, was but partially formed. The nose was perfect; the ossa nasi extending as far up as the junction with the os frontis. The ball of the eye was perfect, but needed the bones that cover it above and on the front side. It had all the voluntary motions, but those of the orbicularis palpebrarum were wanting. The temporal bones were imperfectly formed; the edge that joins the parietal being truncated, parallel with the petrous and mastoid portions. The ear was natural, except in size, which was smaller, by half, than common. The occipital bone extended up to half the distance between the crucial ridge and the foramen magnum. *The cerebrum, cerebellum, and medulla oblongata, were entirely*

*wanting;* and no traces of a tentorium could be discovered. There appeared a slight effort, however, on the part of nature, at the formation of a brain, or rather of its membranes; for two cysts, answering to the right and left hemispheres, and containing a serous fluid, were present. The membranes composing these cysts, were of a fibro-cartilaginous texture. A pair of forceps being introduced through the foramen magnum, a portion of medullary matter was extracted. Three tongues, or appearances of tongues, added much to the peculiarities of this astonishing freak of nature. One appeared to be an elongation of the uvula, gradually enlarging from its origin to the anterior extremity, where it was obtusely rounded. The passage of the posterior nares was obstructed by it. On its superior surface was a thin coat of very fine hair. Another originated from the right side of the mouth, and bore but a faint resemblance to a tongue. The third was natural in form and situation. This infant, as far as I was permitted to examine it, was perfect in every other respect; having the same power of its limbs, that other infants have. It was of a large size and healthy appearance, and lived eight hours after birth; during which time it manifested a fear of falling when handled, and all the usual signs of self-preservation."

